# Spatial organisation in the human mind as a function of the distance between stimuli

**DOI:** 10.1177/17470218241255690

**Published:** 2024-06-18

**Authors:** Hannah Fenwick, Guillermo Campitelli, Alessandro Guida

**Affiliations:** 1 Murdoch University, Murdoch, Western Australia, Australia; 2 Université Rennes 2, Rennes, France

**Keywords:** SPoARC effect, ordinal position effect, serial order coding, spatial coding, positional tagging, spatialisation, working memory, mental whiteboard hypothesis

## Abstract

Studies investigating serial order in working memory have shown that participants from Western cultures are faster at responding to items presented at the beginning of a sequence using their left hand and faster at responding to items at the end with their right hand. This is known as the spatial positional association of response codes (SPoARC) effect. The SPoARC effect provides evidence that recently presented information is spatially organised in the cognitive system along a horizontal axis. This study investigated the flexibility of spatialisation by testing the effect that distance between items presented on a screen has on the magnitude of the SPoARC effect. It was hypothesised that by increasing the distance between items on a screen a larger SPoARC effect would be found. We used three conditions: central, narrow, and wide. In central, four random letters were presented sequentially at the centre of the screen, in narrow the letters were presented from left to right on the screen, wide was the same as narrow but the separation between the letters was larger. Participants consisted of 64 adults aged 18–55 years. Participants were presented with four random letters, followed by single probe letter; participants had to indicate, by pressing a key on a normal keyboard, if the probe had been in the sequence. We analysed the data with multilevel modelling. We found evidence for the SPoARC effect in all three conditions. But no evidence that the effect varied between conditions.

As humans we store and process a vast amount of information in the mind. Knowing how the cognitive system organises and internally represents information is important for understanding how the mind works (Atkinson & Shiffrin, 1968; Baddeley & Hitch, 1974; Cowan, 2001, 2014; Guida et al., 2016; Guida & Lavielle-Guida, 2014; Lashley, 1951; Logan, 2021). According to a research summary publication by McCrink and de Hevia (2018), the human mind has an adaptive tendency for representing the world spatially through the recruitment of space as an organisational scaffold for information processing. This spatial representation occurs not only when observing a spatially organised external environment but when we are presented with information that is not spatially organised, such as items displayed in the centre of a screen (Dehaene et al., 1993; Gevers et al., 2003; Guida, Abrahamse, & van Dijck, 2020; McCrink & de Hevia, 2018; van Dijck & Fias, 2011). How we spatialise information in the mind not only contributes to the understanding of how the mind works but also has translational implications (Guida, Mosinski, et al., 2020). For example, Guida and Campitelli (2019) stated that it can inform strategies on how to disseminate information to the public and can inform on pedagogical principles on how to present information for more effective learning and teaching. To understand how spatialisation occurs, we can explore both the external and internal influences for how we store and access information in the cognitive system (Guida, Abrahamse, & van Dijck, 2020).

When considering the complexity of human cognition, it would be insufficient for us to only be able to store and recall individual pieces of information; rather, it is necessary for us to be able to process, retain, and retrieve information in an ordered and systematic way (Majerus & Oberauer, 2020; Marshuetz, 2005). Serial order coding explains how people can store and retrieve sequences of information in the order it is received (Abrahamse et al., 2014, 2016; Abrahamse & Guida, 2018; Ebenholtz, 1963; Lashley, 1951). According to current dominant theories, serial order coding uses a positional tagging mechanism to keep items ordered (Guida, Abrahamse, & van Dijck, 2020; Henson, 1998; Marshuetz, 2005), which can be understood as a way to encode items to individual mental coordinates. When an item is presented it is “tagged” to a mental position; when the next item is presented, it is then “tagged” to the next mental position; with all the items “tagged” corresponding to the serial order (De Belder et al., 2015; Guida, Abrahamse, & van Dijck, 2020). A visual representation of positional tagging for three letters presented in serial order is shown in Figure 1.

**Figure 1.**
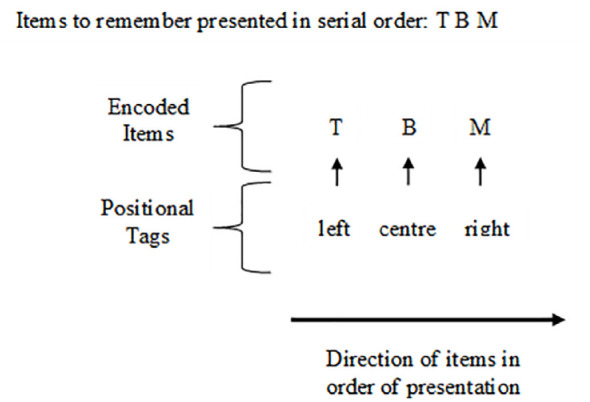
Visual representation for the positional tagging of items in working memory. *Note.* Figure 1 was created by the current authors; however, it is inspired by figures in Guida, Abrahamse, and van Dijck (2020).

In Figure 1, T is presented first in the serial order and is positionally tagged to the left, B is presented next in the serial order and is tagged to the next position (as there is only three items this is the central position), and M is presented last in the serial order and is tagged to the last position on the right. The positional tags in working memory are not actually labelled “left, centre, right” in the mind; this is just to convey the spatial representation. When items are sequentially presented, they are encoded and stored in the required and coherent order, ensuring that when information is retrieved, it is recalled in that same encoded order; this allows for serial order of information to be maintained over time (Botvinick & Watanabe, 2007; Marshuetz, 2005). Without maintaining the serial order of information, we would not be able use language to form sentences, remember list items, or perform most cognitive tasks using memory or reasoning (Majerus & Oberauer, 2020). Consequently, it is essential to better understand the mechanisms involved in serial order coding (Logan, 2021).

As an extension of serial order used for positional tagging, there is further evidence that indicates positional markers are also spatial in nature, with items at the beginning of the serial order being associated with one side of space and items at the end associated with the other (Abrahamse et al., 2014; Guida, Abrahamse, & van Dijck, 2020). This added spatial element in the encoding process can be understood as spatial coding and provides insight into how the cognitive system spontaneously spatialises information when there is no external spatial input (Ginsburg et al., 2014, 2017). The process of serial order and spatial coding is supported by evidence from two main effects: the spatial numerical association of response codes (SNARC) effect and the spatial positional association of response codes (SPoARC) effect (e.g., Dehaene et al., 1993; Gevers et al., 2003; Guida & Lavielle-Guida, 2014; van Dijck & Fias, 2011). Guida and Campitelli (2019) propose that the phenomenon of both the SNARC effect and SPoARC effect is the addition of a spatial element within the cognitive system used to spontaneously organise information along a horizontal or vertical axis internally in the mind.

## SNARC effect

**Figure 2.**
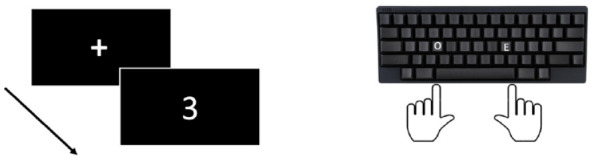
Graphical timeline for one trial of parity judgement task used to elicit a SNARC effect. *Note.* In this example odd “O” is assigned to left-hand responses and even “E” to right-hand responses, the presented digit is “3” which would initiate a faster left-hand response than numbers of higher magnitude.

The SNARC effect is the finding that when completing a memory span task Western participants are faster at responding to smaller numbers with the left hand and faster at responding to larger numbers with the right hand (Dehaene et al., 1993). In a seminal study by Dehaene, et al. (1993), evidence for the SNARC effect was established from multiple experiments conducted using a parity judgement task. As shown in Figure 2, in the first of these studies participants were presented with a single Arabic digit in the centre of a screen and asked to make an odd or even judgement using a keyboard (Dehaene et al., 1993). For half the trials, the left hand was assigned to a key response for “odd” and the right hand was assigned to “even”; the starting assignment was counterbalanced between participants and then reversed between trial blocks. It was found that participants responded faster to numbers smaller in magnitude (e.g., 1, 2, 3, and 4) with their left hand and they responded faster to numbers of larger magnitude (e.g., 7, 8, 9, and 10) with their right hand (Dehaene et al., 1993). Dehaene et al. (1993) termed this finding the spatial-numerical association of response codes effect (SNARC) to highlight the association between spatial position of numbers and numerical order magnitude when responding to single digits. A visual representation of one trial for the task used in Dehaene et al.’s (1993) study is shown in Figure 2.

**Figure 3.**
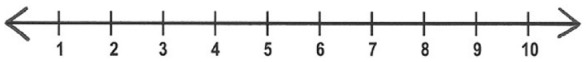
Visual representation of a mental number line from 1 to 10. *Note.* In this representation of a mental number line, smaller numbers are associated with the left and larger numbers are associated with the right side of space. Dehaene et al. (1993) suggest that this is how numbers are retrieved from long-term memory when presented with a digit on the screen.

Dehaene et al. (1993) first explained the SNARC effect with the activation of a mental number line in long-term memory, suggesting that it is due to our familiarity with numbers in order from 1 to 10. These numbers are stored in long-term memory as a number line. In the mental number line, numbers are spatially positioned in order of magnitude as shown in Figure 3.

When presented with a single digit (as in the parity judgement task) the mental number line is activated, and participants are faster at responding with the left hand to low numbers (e.g., 1, 2, 3, and 4) and faster at responding with the right hand to higher numbers (e.g., 7, 8, 9, and 10). This demonstrates that the participant response is associated with where the presented digit is spatially positioned along the mentally represented number line, which is the SNARC effect.

The SNARC effect has been replicated in different conditions; it has been produced irrespective of handedness (the same effect is found in both right-handed and left-handed participants), in conditions where the hands are crossed over, is observed using different variations of the experimental task and found using various ordinal stimuli such as letters of the alphabet and days of the week (Fias et al., 2001; Fischer, 2003; Fischer et al., 2009; Gevers et al., 2003; Nuerk et al., 2011; Wood et al., 2006, 2008; Zhang et al., 2016; Zhou et al., 2020). Interestingly, the SNARC effect is reversed in cultures that read from right to left, follows a vertical direction in cultures that read from top-down, and the effect is not found in people who are illiterate (Hung et al., 2008; Ito & Hatta, 2004; Shaki et al., 2009; Zhou et al., 2020). This finding strongly suggests that default spatial direction is related to cultural reading and writing direction (Göbel et al., 2015; Shaki et al., 2012; Zebian, 2005). Finally, Herrera et al. (2008) found evidence that the SNARC effect disappeared under increased working memory load which raised questions about the validity of Dehaene, et al. (1993) long-term memory account (see also Abrahamse et al., 2016; Previtali et al., 2010; van Dijck et al., 2009). To further investigate the underlying mechanism for the SNARC effect, in 2011, van Dijck and Fias designed a variation of the original SNARC paradigm that led to them finding the SPoARC effect.

## SPoARC effect

**Figure 4.**
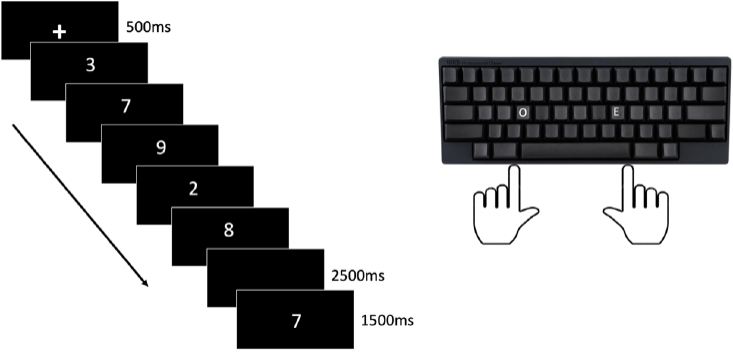
Graphical timeline for one trial of parity judgement task used to elicit a SPoARC effect. *Note.* In this experimental trial odd is assigned to left-hand responses and even to right-hand responses, the presented probe digit is “7” which would initiate a faster left-hand response as it was presented at the beginning of the sequences, irrespective of its magnitude being spatially associated to the right side of a mental number line, as suggested in the long-term memory account for SNARC.

The SPoARC effect provides evidence that recently presented information is spatially organised in the cognitive system along a horizontal axis. Rather than presenting participants with a single digit, in van Dijck and Fias’ 2011 study, participants were presented with random sequences of 5 digits to memorise. As shown in Figure 4, the digits were presented one after the other in the centre of a screen. Following the sequence, participants were presented with a single probe digit and asked to make an odd or even judgement using a keyboard, but only if that probe had been in the sequence. Assignment of odd and even to left- and right-hand responses was split between trial blocks and reversed and counterbalanced across participants (van Dijck & Fias, 2011).

Participants were found to respond faster to a probe that had been presented at the beginning of the sequence with their left-hand and were faster to respond to a probe presented later in the sequence with their right hand. This association between spatial response effect and ordinal position of items is the Spatial Positional Association of Response Codes (SPoARC) effect (Guida, Abrahamse, & van Dijck, 2020). As with SNARC, the SPoARC effect is robust across varying experimental conditions such as crossed hands and audio presentation and is found with different stimuli such as: letters, words, pictures, symbols, shapes, and coloured dots (Bottini et al., 2016; Darling et al., 2020; Ginsburg et al., 2017; Guida & Porret, 2022; Shi, Wang, Deng, & Xu, 2020; Shi, Wang, Zhao, & Zhao, 2020; Wang et al., 2019). The SPoARC effect has also received support in eye-tracking studies (Hartmann, 2015; Hartmann et al., 2021; Rinaldi et al., 2018).

**Figure 5.**
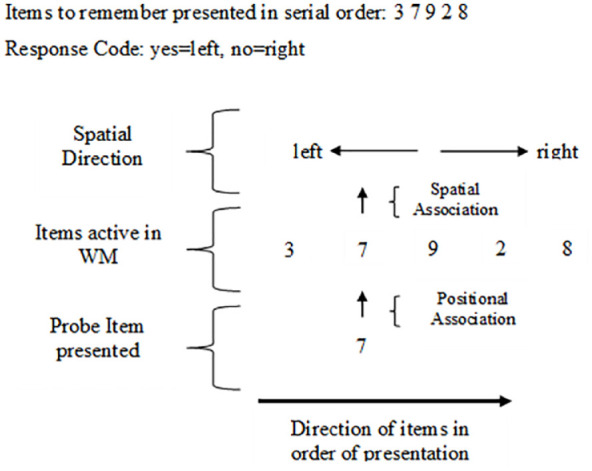
Visual representation of items spatially ordered in working memory for the SPoARC effect showing the spatial and positional associations. *Note.* This figure shows the presented sequence active in WM in its serial order. When the Probe 7 is presented, it is associated to the position of the seven from the sequence, which in this trial is on the left side of space; thus, it would elicit a faster left-hand response for the yes response as it is associated with the left side of space.

To account for the SPoARC effect, van Dijck and Fias (2011) presented a working memory account suggesting both the SNARC and SPoARC effects could be explained by underlying working memory processes. Considering that the mental number line hypothesis for the SNARC effect relies on the storage and activation of information stored in long-term memory it was established that random sequences of numbers (or other stimuli) could not be retrieved from long-term memory, and thus, the account that was used to explain the SNARC effect cannot be used to explain the SPoARC effect. Instead, van Dijck and Fias (2011) proposed that as each item is presented, it is spatially coded in order of presentation in working memory. As with the SNARC effect, the default spatial coding and positioning of items in working memory is thought to correspond to cultural reading and writing direction (Guida et al., 2018). In the initial study by van Dijck and Fias, participants were Westerners; therefore, items were spatially coded from a left-to-right direction. As depicted in Figure 5, for the working memory account, it is thought that participants hold the presented sequence in working memory, spatially organised in order from left-to-right. When the probe digit is presented, faster left-hand responses are recorded when they are associated to the items that are spatially represented on the left (which are the items that had been presented first) and faster right-hand responses are recorded when they are associated to items spatially represented on the right (which are the items that were presented last) (Abrahamse et al., 2016).

## Unified theoretical accounts for both SNARC and SPoARC

**Figure 6.**
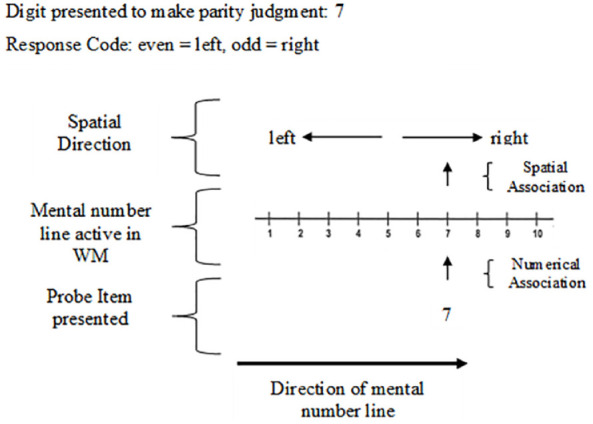
Visual representation of the working memory account for SNARC effect showing the spatial and numerical associations. *Note.* Figure 6 shows the mental number line active in WM, when the Probe 7 is presented, it is associated to the position of the seven in the mental number line which is on the right side of space; therefore, it would elicit a faster right-hand response for odd judgement as it is associated to right side of space.

For the working memory account to explain the SNARC effect, van Dijck and Fias (2011) proposed that rather than the activation of a mental line purely as a long-term memory process, the information stored in long-term memory is transferred to working memory, with a mental representation of items spatially organised from left-to-right in the same order they are stored in long-term memory. As shown in Figure 6, with numbers 1–10, the mental number line stored in long term memory would be transferred to working memory with smaller numbers coded to the left side of space and larger numbers coded to the right side of space (van Dijck et al., 2009; van Dijck & Fias, 2011).

Thus, the working memory account can explain both SNARC and SPoARC, whereas a long-term memory account could only account for SNARC. The working memory account can also explain the SNARC effect disappearing under working memory load as found in Herrera et al. (2008). When working memory load is added to a task, it limits or overrides the capacity for further items stored in long-term memory to be transferred to working memory. Therefore, there is minimal or no effect that favours either left- or right-hand responses found when working memory load is added. If the SNARC effect was purely a long-term memory process, then adding working memory load should not override the SNARC effect (van Dijck & Fias, 2011; van Dijck et al., 2009).

Building on van Dijck and Fias’ (2011) working memory account, Abrahamse et al. (2014) have presented another account; the mental whiteboard hypothesis, which can explain both SNARC and SPoARC and provide further insight for understanding spatialisation using positional tagging. The mental whiteboard hypothesis describes a metaphorical “whiteboard” where information from long-term memory and incoming information can be displayed, manipulated, and encoded (Abrahamse et al., 2014). In line with models of serial order coding using positional tagging, the mental whiteboard hypothesis supports the idea that each incoming item presented in a sequence is associated with a positional marker or “tag” depending on its ordinal position in the sequence and that these positional markers are spatial in nature (Guida, Abrahamse, & van Dijck, 2020). It also suggests that the cognitive system internally creates a spatial template where incoming items can be bound to mental coordinates and thus can be used to retain spatial information (Abrahamse et al., 2014). According to the proponents of the mental whiteboard hypothesis, space is flexibly recruited for serial order coding dependent on the task at hand, and therefore, can be used to explain variations in experimental designs eliciting either a SNARC or SPoARC effect (Abrahamse et al., 2014).

For SNARC, when information is transferred from long-term memory, it is associated with spatial position markers from the ordinal position it was encoded with. For example, a SNARC task using numbers would require the mental number line to be displayed on the mental whiteboard, which also provides the spatial template for positional tagging (Abrahamse et al., 2014). As it is only necessary for information from long term memory that is relative to the task at hand to be activated on the mental “whiteboard,” this can better explain why in some tasks the same stimuli can be represented spatially on the left and in other tasks the same stimuli are spatially represented on the right. This is because different stimuli sets have different parameters which would be associated with different position markers on the whiteboard, and this would determine if they were mentally represented on the left or right side of space (Dehaene et al., 1993; van Dijck et al., 2009). In reference to SPoARC, activation of a spatial template on the mental whiteboard would allow for items to be spatially tagged as they enter working memory (Guida, Abrahamse, & van Dijck, 2020). Although similar to the original working memory account, the proposal of a mental whiteboard provides an account for variations in spatialisation and is the dominant theory for recent SPoARC studies (Guida & Porret, 2022).

In 2019, Guida and Campitelli conducted a review of SNARC and SPoARC studies highlighting the flexible nature of spatialisation and proposed three strategies for how the cognitive system prioritises the organisation of information in the mind. First, if spatial information is provided in the environment, then this spatial information will be used for ordering incoming items. Second, if there is no spatial information provided, then the spatial information related to how the items are stored in long-term memory will be utilised (this would be the case for SNARC like tasks where the activation of a mental number line already holds the spatial positioning). Third, if there is no spatial information provided either in the environment or in long-term memory (such as with the presentation of random sequences, placing items one at a time in the centre of a screen), then incoming information will be spatialised in order of presentation consistent with default spatial coding associated to cultural reading and writing direction (this would be the case for SPoARC-like tasks). These parsimonious strategies emphasise that the cognitive system will follow the most economical or efficient process for encoding and retrieving information (Guida & Campitelli, 2019; Guida, Abrahamse, & van Dijck, 2020).

Although, as indicated above, the second and third strategies can be associated with previous findings from SNARC and SPoARC, evidence for the first strategy has come from more recent research that manipulated the external input using SPoARC-like paradigms (Guida, Abrahamse, & van Dijck, 2020; Guida et al., 2016; Guida, Mosinski, et al., 2020). In a 2020 study, Guida, Mosinski, et al. investigated if the spontaneous direction of spatialisation could be reversed by instructing participants to memorise information in a right-to-left direction, incongruent to their usual left-to-right reading direction (Guida, Mosinski, et al., 2020). Participants were auditorily presented with lists of five consonants at a rate of 3 seconds per item, followed by a random probe. As with other SPoARC paradigms, participants were asked to indicate if the probe had been in the sequence using left-handed or right-handed keyboard responses. What they found was by asking participants to mentally organise items from right-to-left, a reverse SPoARC effect was observed. This suggests that participants used the task instruction as an environmental cue for spatial direction when encoding the presented sequences. To determine if the magnitude of the reversed SPoARC effect was comparable with a regular SPoARC effect, they compared the results of the Guida, Mosinski, et al. (2020) study with the results of a previous study that followed the same procedure; except, in the comparison study by Guida et al. (2016), participants had not been instructed to memorise the sequences in a right-to-left direction. The results showed no significant difference in magnitude for the regular and reversed SPoARC effects.

**Figure 7.**
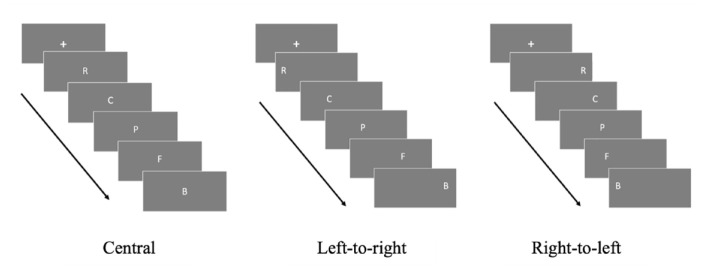
Graphical timelines showing stimuli presentation for one trial in each condition. *Note.* Figure 8 is not from the original study, it has been included to provide a visual depiction for the three different external presentation conditions used in Guida, Abrahamse, and van Dijck (2020).

This provides evidence that, depending on the task at hand, individuals can reverse or override the spontaneous default cultural direction of spatialisation with no added cognitive cost (Guida, Mosinski, et al., 2020). It also supports the flexibility of space being recruited for serial order coding to best fit the task at hand as proposed in the mental whiteboard hypothesis (Abrahamse et al., 2014). In this instance, the task at hand instructed right-to-left organisation; thus, items were spatially coded in order in a right-to-left direction. This finding is consistent with the first strategy proposed by Guida and Campitelli (2019) that as a first priority for organisation space will be recruited from the environment. Further evidence for external influence for spatialisation was established with Guida, Abrahamse, and van Dijck (2020) study manipulating the external presentation direction of items presented on a screen either: centrally, left-to-right or in a right-to-left direction (Figure 7).

Based on the mental whiteboard hypothesis, it was predicted that due to the flexible nature of the cognitive system, in the central and left-to-right presentation condition a regular SPoARC effect would be found, whereas in the right-to-left presentation condition a reversed SPoARC effect would be found (Abrahamse et al., 2014; Guida, Abrahamse, & van Dijck, 2020). Using a standard SPoARC paradigm with five letter sequences a regular SPoARC effect was found in the central and left-to-right presentation and a reversed SPoARC effect was found in the right-to-left presentation with no indication of cognitive cost for the reversed effect. More recently, Wang et al. (2022) also indicated that their results were compatible with Guida and Campitelli’s (2019) parsimonious principles.

## Overview of the current study: manipulating external presentation distance using a SPoARC paradigm

The current study further explores the internal and external interplay of spatialisation following a similar paradigm to Guida, Abrahamse, and van Dijck (2020). Specifically, this study aims to investigate the flexibility of spatialisation by testing the effect that distance between spatial presentation on a screen has on the SPoARC effect. We used three conditions: the central condition, narrow condition, and the wide condition. Based on Guida, Abrahamse, and van Dijck (2020), for all the three conditions, it is expected that there will be a SPoARC effect; determined by a difference in reaction time in relation to the response hand (left-hand vs. right-hand) and the position in the sequence that the probe item was associated with (Positions 1, 2, 3, and 4). For the probe items that are presented corresponding to the beginning of the sequence, it is thought that there will be a faster reaction time for left-hand responses and for the probe items that correspond to presentation at the end of the sequence, there should be a faster response time from the right hand. Indicative of a classic SPoARC effect, there should be an inverse relationship between reaction time differences for hand of response and position in the sequence. The central condition has been included as a control condition to ensure that a genuine SPoARC effect can be obtained within our sample.

It is also expected that there should be a difference in the magnitude of the SPoARC effect when comparing the narrow and wide conditions. We hypothesised that there will be a larger effect found in the wide condition over the narrow condition. We hypothesised that the distance between stimuli on the screen should determine the spatial coordinates of the memoranda on the spatial template activated in working memory for encoding (see Guida et al., 2016; Guida & Lavielle-Guida, 2014; Guida & Maherault, 2021; Majerus & Oberauer, 2020; Melton, 1963; Roediger Iii & Guynn, 1996). We hypothesised that in the central condition the default spatial template congruent with left-to-right reading direction will be activated for positional tagging of items left-to-right. In the narrow condition, we predicted that a spatial template using the spatial coordinates of the items on the screen will allow for positional tagging narrowly from left-to-right. In the wide condition we predicted that by manipulating item presentation to increase the space between items displayed on the screen, larger spatial coordinates will be used on the spatial template activated in working memory to allow for a wider spatial distance between positional tags than in the narrow presentation condition.

## Methods

### Participants

To determine the number of participants, a power analysis from Guida, Abrahamse, and van Dijck (2020) was used. The SPoARC slope, which is the core of the statistical analysis on the SPoARC effect, is run via a regression analysis for repeated measures of the reaction times differences obtained when subtracting the left-hand reaction times from the right-hand reaction times for each sequence position. A two-tailed *t*-test is then performed to evaluate whether the obtained regression slopes significantly deviated from zero. If we assume a two-sided criterion for detection and a type I error of α = .05, to reach a power of 80%, a sample of 34 participants is needed to detect an effect size of Cohen’s *d* ⩾ .50 (see Hartmann et al., 2021). A total of 64 participants were recruited to participate; 49 undergraduate psychology students were awarded 1 credit point for participation. The remaining 15 participants were recruited through research poster placement on campus and approved social media platforms. The participant sample comprised of 76.56% females (23.44% males), aged between 18 and 55 years, (*M* = 24.98, *SD* = 7.41).

### Materials

Stimuli consisted of a pool of 18 consonants, this included all consonants minus “L,” “W,” and “Z.” The “L” was excluded as it was one of the response keys for the task. The “W” was excluded as it contains multiple syllables which could influence articulatory rehearsal and recall time (see Longoni et al., 1993). Finally, the letter “Z” was excluded to ensure an even number of consonants after the removal of “L” and “W,” with “Z” being deemed a more obscure letter (Guida, Abrahamse, & van Dijck, 2020). Stimuli appeared in font type Open Sans, was approximately 3 cm × 3 cm on screen. All font was displayed in white on a light grey background. The screen size was 30 cm × 53 cm, with a resolution of 1,680 x 1,050 pixels. The experiment was built using PsychoPy 2 builder software (Peirce et al., 2019).

### Task

In the central condition, there were a total of 56 experimental trials. Each trial consisted of the presentation of a random four-letter sequence followed by presentation of a single probe consonant in the centre of the screen. As depicted in Figure 5, for the central condition, items presented were all centrally placed, with all stimulus items presented in the same place, one at a time, one after the other on the screen; the central screen position corresponded to a visual angle of 0.0°.

In the narrow condition, there were 48 trials. Each trial consisted of the presentation of the random four-letter sequence from left-to-right, followed by presentation of a single probe consonant in the centre of the screen (only four items were used as there is no preference for left- or right-hand responses for central positioning either spatially or ordinally for a middle item when using five items). For the narrow condition, items were spaced in order of presentation in a left-to-right direction across the screen, with the first item presented partially to the left at 6.23° left of centre. The second item was presented between where the first item had appeared and the centre of the screen at 2.08° left from centre. The third item was presented the same distance from centre as the second item but right of centre by 2.08°. The last item in the sequence presented partially to the right equal distance from centre as the first item, 6.23° to the right of centre.

As with the narrow condition, there were also 48 trials in the wide condition. Stimuli were presented from left-to-right equally across the entire width of the screen with the first item presented 23.58° left of centre, Item 2 presented 8.28° left of centre, Item 3 presented 8.28° right of centre, and Item 4 presented 23.58° right of centre.

For each trial, there were two possible responses (yes or no). Half the participants started with “A”= yes, “L” = no, with the other half starting with the reverse instruction “L”= yes, “A” = no. In each of the three conditions, there were two separate blocks of trials, to allow for counterbalancing and the reversal of the response keys across participants and within each condition.

**Figure 8.**
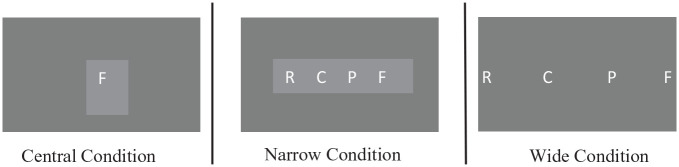
Visual representation for stimuli position for central, narrow, and wide conditions. *Note.* This figure visually represents the spatial distance between presentation positions for each individual condition and therefore all the items in the sequence are shown on the same screen; in the experiment, only one letter appeared at a time and as such is overlapping.

The sequences were pseudo-randomly generated from the specified pool of consonants. There was an equal number of trials with “yes” probes and “no” probes (“yes” indicating that the probe in that trial was in the sequence and “no” indicating that the probe was not in that the sequence for that trial). The presentation positions were equally probed (the same number of probes appeared equally in Positions 1, 2, 3, and 4). In the central presentation condition, each position was probed 7 times; for this condition the probes were only equal at a group level as half the participants had two more probes in Positions 2 and 3 and the other half of participants had two more positions probed in Positions 1 and 4 (this was due to the division of consonants between the positions probed). For both, the narrow and wide conditions each position was probed 6 times for each individual participant. Figure 8 shows the different screen placement of stimuli between conditions.

### Procedure

Ethics approval was granted by the Murdoch University Human Research Ethics Committee. Participants provided consent and completed a memory task on a computer under the observation of the experimenter. Participants were instructed to remain 55 cm from the screen throughout the experiment. All participants began with the central condition. At the beginning of the first block there were four practice trials, followed by 28 test trials. The key response instructions were reversed for the next block consisting of another four practice trials and another 28 test trials. This completed the two blocks of trials for the central condition. Half the participants started the second block with the narrow condition and the other half with the wide condition, this was then reversed. The narrow and wide conditions were broken into two separate blocks each starting with four practice trials followed by 24 test trials.

**Figure 9.**
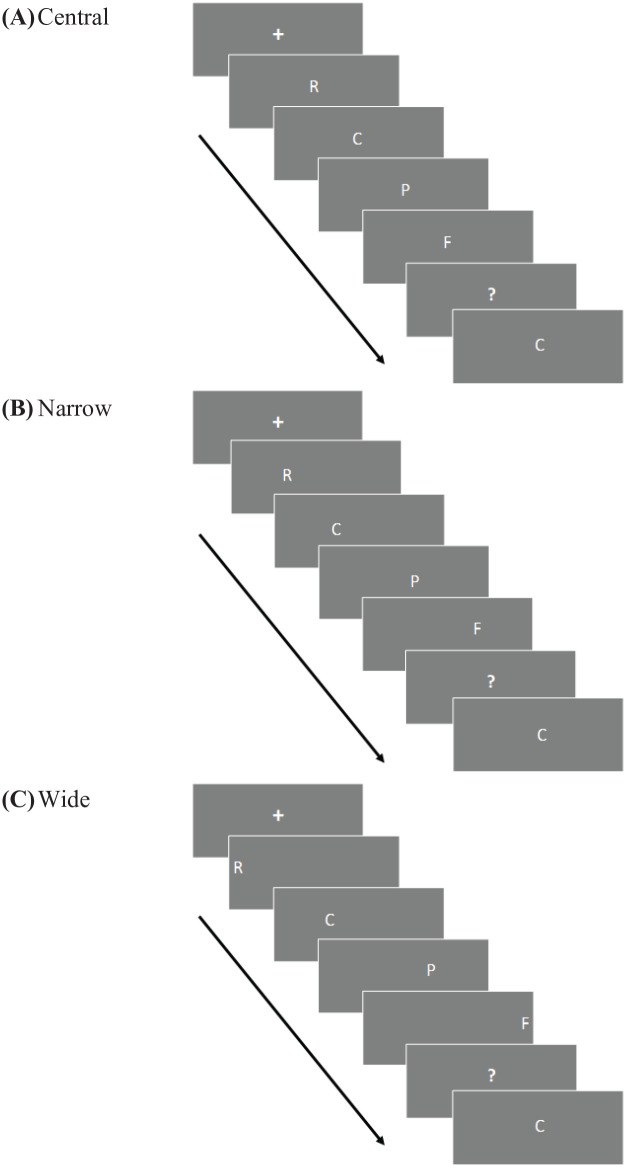
Graphical timeline for one trial in each condition. *Note.* The blank screens that appear between stimuli have been removed for this figure.

All trials began by participants pressing the “spacebar” on the keyboard. This was followed by presentation of a fixation cross “+” in the centre of the screen indicating the sequence was about to be presented. The fixation cross was presented for 500 ms followed by 500 ms of a blank screen. After the fixation, the sequence was presented, one letter was presented for 2,200 ms followed by a blank screen for 800 ms of for the central condition and one letter was presented for 800 ms followed by blank screen for 200 ms in the narrow and wide conditions. This was followed by the presentation of “?” for 250 ms in the centre of the screen; the probe then appeared until the participant made a keyboard response. After the response was made, feedback was displayed on the screen, indicating if the response was correct or incorrect. This completed one trial. The entire experiment took approximately 45–50 min to complete.^1^ Figure 9 shows a graphical timeline for one trial of each condition.

### Data analysis

We used a repeated measures research design, in which all the participants went through the three conditions. The independent variable (IV) was the position of stimulus on the screen. The assignment for response hand (left hand = yes, right hand = no) was manipulated within each condition. The dependant variables (DV) were the accuracy of responses (either correct or incorrect) and response time recorded in milliseconds. The first analysis obtained mean reaction times (RT) and accuracy of responses. All “no” responses, incorrect responses, and RT lower than 100 ms were eliminated from the data set (see Ratcliff, 1993). Responses more than 2.5 standard deviations above and below mean RT were eliminated (The 2.5 *SD* limit for these outliers were calculated within participant, in each position, per hand). In addition, one remaining outlier of more than 7,000 ms was removed (the removal of this outlier did not impact the findings or the overall conclusions of this study). Separate data frames were created for each of the conditions and analysis run to obtain descriptive statistics for RT in each individual condition, for each position, per hand. Following Guida, Abrahamse, and van Dijck (2020) to account for the skewness of data from RT each data set was Log transformed and the same analyses were performed for Log RT data (see also Lo & Andrews, 2015).

Consistent with the standard procedure for SPoARC as outlined in Guida, Abrahamse, and van Dijck (2020) two steps are necessary to determine a SPoARC effect: first, a regression analysis is conducted with Log RT as the DV, and position and hand as IV; second, if the “hand of response x position” interaction is found, a separate analysis is run with Log difference reaction times (dRT) as the DV and Position as the IV (Guida, Abrahamse, and van Dijck 2020; Hartmann et al., 2021). For this study, analyses used multilevel modelling with observations set as Level 1 and participants set at Level 2 (using this approach was aimed at avoiding violating assumptions of independence and sphericity) (see Hoffman & Rovine, 2007).

For the first analysis, we performed multilevel modelling for the Log RT data frames with four nested multilevel models estimated: Model 0, had only the intercept as a fixed effect and random effect; Model 1, intercept plus hand as fixed effects, intercept as random effect; Model 2, intercept plus hand, plus position as fixed effects and intercept as a random effect; Model 3, intercept plus the interaction of “Hand × Position” as fixed effects and intercept as the random effect. We assessed models using the Akaike’s information criterion (AIC) and the Bayesian information criterion (BIC). Both the AIC and BIC are useful for model selection and use different parameter penalties for each free parameter used in the model to avoid overfitting (see Kuha, 2004). The BIC is more conservative, providing more penalties for each parameter, over the AIC.

For the second analysis, multilevel modelling was performed on the Log RT differences obtained by subtracting left-hand RT from right-hand RT for each position. Each participant’s dRT was regressed for each corresponding sequence position. In this situation, a typical SPoARC effect would be represented by a negative regression slope; in other words, the dRT should favour the left hand in the initial positions and gradually lose this advantage moving to the next position. For the analysis using Log dRT, three nested multilevel regression models were estimated: Model 0 had only the intercept as a fixed effect and a random effect; Model 1, intercept plus position as a fixed effect and intercept as a random effect; Model 2, intercept plus position as fixed effects and the interaction of the intercept plus position as a random effect.

Finally, to determine whether there were any differences between conditions a comparison of the overall magnitude of each effect was performed. This was done by running a regression coefficient comparison analysis to determine any evidence for differences in effects with an alpha level of .05 (see Clogg et al., 1995).

## Results

The overall accuracy for correct trial responses was 93.8% (*SD* = 24.1%). This was comparable with accuracy for each of the conditions; central (94.0%, *SD* = 23.8%), narrow (93.8%, *SD* = 24.2%), and wide (93.7%, *SD* = 24.4%). The rest of the analyses from here on only used trials in which both a “correct” and a “yes” response was made. Mean scores and standard deviations for RT split by condition are shown in Table 1.

**Table 1.** Mean scores for reaction time for hand of response in each position split by condition.

Position	Hand	*M*	*SD*	95% CI	
				LL	UL
Central condition					
1	Right	1,133	425	1028	1,237
	Left	1,017	395	920	1,113
2	Right	1,180	492	1060	1,301
	Left	1,150	467	1035	1,264
3	Right	1,109	372	1017	1,200
	Left	1,094	401	995	1,192
4	Right	1,097	439	987	1,202
	Left	1,121	404	1021	1,220
Narrow condition					
1	Right	1,079	422	976	1,182
	Left	1,004	484	886	1,123
2	Right	1,062	385	968	1,156
	Left	1,050	413	949	1,151
3	Right	992	402	894	1,091
	Left	1,065	475	949	1,182
4	Right	967	356	879	1,091
	Left	1,047	402	948	1,145
Wide condition					
1	Right	1,093	431	987	1,199
	Left	978	375	886	1,070
2	Right	1,111	421	1008	1,213
	Left	1,018	337	935	1,100
3	Right	1,071	455	960	1,183
	Left	1,017	387	923	1,112
4	Right	1,055	396	958	1,152
	Left	1,072	399	974	1,170

*Note.* M = mean, SD = standard deviation, CI = 95% confidence intervals; LL = lower limit UL = upper limit, reaction time in milliseconds.

**Figure 10.**
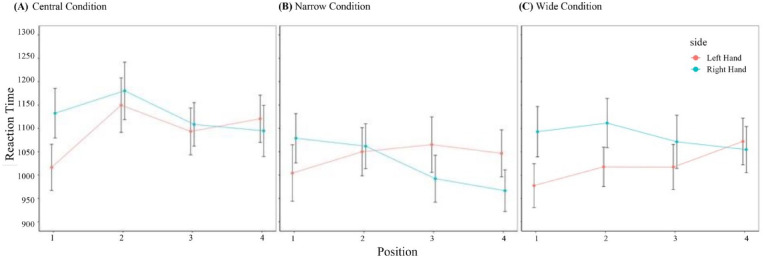
Mean reaction times for left-hand and right-hand response in each position split by condition. *Note.* Error bars show Standard Error of the Mean, reaction time in milliseconds.

A visual representation of the results using log transformed data can be observed in Figure 10, which allows us to observe a crossover of RT between left hand and right hand from Position 1 to Position 4 in the three conditions.

For the first SPoARC analysis, to assess if there was an effect for hand of response and position in the sequence multilevel modelling was performed using Log RT data for each condition. The results for multilevel modelling in the central condition are shown in Table 2. As shown in Table 2, using the AIC, Model 3 best fit the data (AIC = 917.57); however, using the BIC, Model 0 best fit the data (BIC = 940.44). Thus, to further compare Model 0 and Model 3, a chi-square difference calculation was performed. We found evidence in favour of Model 3, χ^2^(3) = 12.67, *p* = .005.

**Table 2.** Multilevel modelling of log reaction time for central condition.

Parameter	Model 0		Model 1		Model 2		Model 3	
	Estimate	*SE*	Estimate	*SE*	Estimate	*SE*	Estimate	*SE*
Fixed effects								
Intercept	6.922	0.038	6.907	0.039	6.883	0.042	6.842	0.045
Hand			0.030	0.01478	0.029	0.015	0.115	0.036
Position					0.010	0.007	0.026	0.009
Hand × Position							-0.034	0.013
Random effects								
Intercept	0.090		0.090		0.090		0.090	
Residual	0.090		0.090		0.090		0.090	
Fit statistics								
Parameters	3		4		5		6	
Deviance	918.21		914.17		912.09		905.57	
AIC; BIC	924.21; 940.44		922.17; 943.80		922.09; 949.13		917.57; 950.02	
χ^2^ difference			4.05		2.08		6.52	

*Note.* SE, Standard error; AIC, Akaike information criterion; BIC, Bayesian information criterion; χ^2^, chi-square differences. The values for Random effects are variances. Hand × Position refers to the interaction.

In the narrow condition, multilevel modelling is shown in Table 3. As shown in Table 3, using the AIC, Model 3 best fit the data (AIC = 931.81); however, using the BIC, Model 0 best fit the data (BIC = 958.59). Thus, to further compare Model 0 and Model 3, a chi-square difference calculation was performed. We found evidence in favour of Model 3, χ^2^(3) = 16.99 *p* < .001.

**Table 3.** Multilevel modelling of log reaction time for narrow condition.

Parameter	Model 0		Model 1		Model 2		Model 3	
	Estimate	*SE*	Estimate	*SE*	Estimate	*SE*	Estimate	*SE*
Fixed effects								
Intercept	6.835	0.040	6.842	0.407	6.859	0.047	6.79	0.048
Hand			-0.014	0.017	-0.015	0.017	0.132	0.041
Position					-0.007	0.007	0.022	0.011
Hand × Position							-0.058	0.015
Random effects								
Intercept	0.097		0.097		0.097		0.097	
Residual	0.098		0.098		0.098		0.097	
Fit statistics								
Parameters	3		4		5		6	
Deviance	936.81		936.02		935.18		919.81	
AIC; BIC	942.81; 958.59		944.02; 965.07		945.18; 971.49		931.81; 963.38	
χ^2^ difference			0.78		0.84		15.37	

*Note. SE*, Standard error; AIC, Akaike information criterion; BIC, Bayesian information criterion; χ^2^, chi-square differences. The values for Random effects are variances. Hand × Position refers to the interaction.

In the wide condition, the result for the multilevel modelling is shown in Table 4. As shown in Table 4, using the AIC, Model 3 best fit the data (AIC = 668.98); however, using the BIC, Model 1 best fit the data (BIC = 696.59). Thus, to further compare Model 1 and Model 3, a chi-square difference calculation was performed. We found evidence in favour of Model 3, χ^2^(2) = 7.89, *p* = .019.

**Table 4.** Multilevel modelling of log reaction time for wide condition.

Parameter	Model 0		Model 1		Model 2		Model 3	
	Estimate	*SE*	Estimate	*SE*	Estimate	*SE*	Estimate	*SE*
Fixed Effects								
Intercept	6.862	0.040	6.841	0.041	6.828	0.044	6.774	0.047
Hand			0.041	0.015	0.041	0.015	0.150	0.038
Position					0.005	0.007	0.027	0.010
Hand × Position							-0.043	0.014
Random effects								
Intercept	0.099		0.099		0.099		0.099	
Residual	0.082		0.081		0.081		0.081	
Fit Statistics								
Parameters	3		4		5		6	
Deviance	674.92		667.60		667.02		656.98	
AIC; BIC	680.92; 696.66		675.60; 696.59		677.02; 703.25		668.98; 700.46	
χ^2^ difference			7.32		0.58		10.04	

*Note. SE*, Standard error; AIC, Akaike information criterion; BIC, Bayesian information criterion; χ^2^, chi-square differences. The values for random effects are variances. Hand × Position refers to the interaction.

For the second SPoARC analysis, reaction time differences were obtained by calculating mean scores for right-hand minus left hand. Mean difference scores for RT with standard error and 95% confidence intervals are shown in Table 5.

**Table 5.** Mean difference scores for reaction time split by condition.

Position	*M*	*SE*	95%*CI*	
			LL	UL
Central condition				
1	110.14	52.85	6.54	213.73
2	30.61	64.16	-95.15	156.36
3	14.96	41.69	-66.75	96.67
4	-25.81	54.51	-132.65	81.04
Narrow condition				
1	74.60	54.96	-33.13	182.33
2	4.81	49.88	-92.96	102.58
3	-72.92	43.98	-159.13	13.29
4	-98.73	48.20	-193.20	-4.26
Wide condition				
1	120.78	46.95	28.77	212.80
2	93.69	44.88	5.71	181.66
3	54.04	47.36	-38.79	146.87
4	-17.50	41.12	-98.10	63.09

*Note. M* = mean, *SE* = standard error, *CI* = 95% confidence intervals [lower, upper] limits,

Reaction time in milliseconds. Reaction time differences calculated using mean scores for right hand minus left hand.

**Figure 11.**
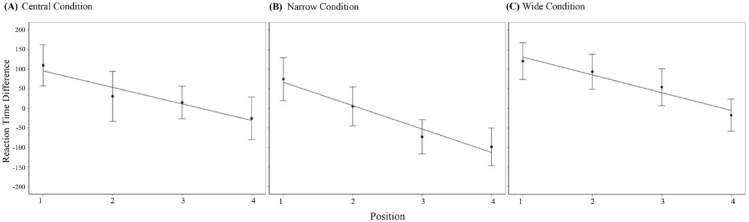
Regression lines showing mean difference in reaction times for position split by condition. *Note.* Error bars show standard error of the mean. dRT in milliseconds.

Mean dRT showing regression lines for position split by condition is shown in Figure 11.

To determine the best fit for dRT for each position, multilevel modelling was run with Log dRT as DV and Position as IV. (for the complete analysis see Supplementary Material; the datasets generated during and/or analysed during the current study are available in the Open Science Framework repository, https://osf.io/nymg8/). In all three conditions, the model including position as Fixed Effects best fit the data using both AIC and BIC.

Finally, a regression coefficient comparison was performed to determine any differences between the conditions. We did not find evidence in favour of differences among conditions: central versus narrow (*z* = 1.142, *p* = .253); central vs. wide (*z* = 0.188, *p* = .851); narrow vs. wide (*z* = -0.966, *p* = .334).

## Discussion

Two hypotheses were tested; first, that there would be a SPoARC effect in all three conditions, and second, that the SPoARC effect would be greater in the wide condition in comparison with the narrow condition. In support of the first hypothesis, there is strong evidence of a SPoARC effect in all three conditions. Contrary to our second hypothesis, we found no evidence in favour of differences in the magnitude of the SPoARC effect between the narrow and wide conditions. The results from the SPoARC analysis in each condition are comparable with previous studies. In the central condition we found an average decrease of 45.32 ms per position compared with 35.9 ms per position reported in the central condition for Guida, Abrahamse, and van Dijck (2020). In the narrow condition we found a difference of 57.78 ms per position and in the wide condition there is a difference of 45.98 ms, both comparable with the left-to-right condition in Guida, Abrahamse, and van Dijck (2020), in which they found a decrease of 37.5 ms per position. This finding adds support to the robust findings for the SPoARC effect, demonstrating that faster left-hand responses are elicited for items that are presented at the beginning of a sequence and faster right-hand responses are elicited for items presented at the end of a sequence. This also provides support to existing evidence for the involvement of a spatial element in serial order coding. However, the lack of evidence of difference in the magnitude of the SPoARC effect between conditions is not consistent with the predictions based on the mechanisms for positional tagging.

The proponents of the mental whiteboard hypothesis suggest that spatial coordinates or templates are recruited to organise information using positional tagging as best fits the task at hand (Abrahamse et al., 2014). To formalise what constitutes “best fits the task” we introduced Guida and Campitelli’s (2019) three parsimonious strategies for spatialisation: first, if available, spatial information from the environment will be used. Second, if no spatial information is provided, the spatial information from long-term memory will be utilised. Third, if no spatial information is provided in the environment or in long-term memory, incoming information will be spatialised in accordance with the default cultural reading and writing direction (Guida & Campitelli, 2019).

Based on the previous results from Guida, Abrahamse, and van Dijck (2020) that found no cognitive cost for a reverse SPoARC effect, it was suggested that in line with both the mental whiteboard hypothesis and the proposed strategies for how we spatialise information, the central condition recruited a default spatial template for serial order coding consistent with the third strategy. Whereas a spatial template using the external presentation direction was recruited for encoding items in working memory in both the left-to-right and right-to-left conditions consistent with the first strategy (Guida, Abrahamse, & van Dijck, 2020). Following on from this, in the current study we predicted that, in the central condition a default spatial template following reading direction would be activated in working memory to allow for positional tagging of each item left-to-right, as per the third strategy, to induce a SPoARC effect. For the narrow and wide condition, it was assumed that the first strategy would be employed with a spatial template containing the different spatial distances between letters on the screen for each condition being activated in working memory.

As mentioned by one reviewer, one possible account for the lack of difference between the narrow and wide conditions could be that the SPoARC effect may be a postencoding effect whereby temporally encoded items are recoded using more abstract spatial codes during maintenance and/or retrieval. In other words, participants could encode the items using a temporal tag (e.g., Brown et al., 2007; Brown et al., 2000), and it would be only during the maintenance phase or at retrieval that the temporal value of the tags is transformed into a spatial value (to wit, spatialisation), with the first temporal tags being linked with left and the lasts with the right following our left to right writing/reading system. Given that there was no temporal difference at encoding between the narrow and the wide conditions in our experiment (the presentation rate was identical), it would then be logical that we found no effect in terms of spatialisation, as identical temporal tags would be transformed into identical spatial tags (at maintenance or at retrieval). Although this is definitely a possibility that we cannot discard in this experiment, it is important to keep in mind that results from Guida, Abrahamse, and van Dijck (2020) were not compatible with this hypothesis. In their study, the authors presented their stimuli from left to right and from right to left. The latter had the advantage of opposing a temporal tagging hypothesis and a direct spatial hypothesis (i.e., the mental whiteboard hypothesis). If one imagines “G,” “B,” “F” as a sequence entering WM and displayed from right to left then a direct spatial hypothesis would predict that “G” is linked with rightmost spatial value, “B” to the middle and “F” to the extreme left. Conversely, in a temporal tagging hypothesis, participants should first temporally tag the items and only after spatially tag them. Given our example, and since “G” is the first item, it should acquire the leftmost spatial value (in western cultures), “B” a middle spatial value and “F” an extreme right value, that is, the opposite outcome of the direct spatial hypothesis. Guida, Abrahamse, and van Dijck (2020) observed a result against the temporal tagging hypothesis.

Another possibility to account for the results is that as all three conditions were exposed to the central condition first the default spatial template remained activated for the narrow and wide conditions. This would avoid adding to the extra cognitive cost required to manipulate the spatial template which would have needed to be activated to incorporate the different external spatial coordinates from the different spatial distances on the screen.

Evidence from the literature has shown that when items in working memory are presented congruent to reading and writing direction there is an increase in performance (see Fischer-Baum & Benjamin, 2014; McCrink & Shaki, 2016). In discussing the findings in Guida, Abrahamse, and van Dijck (2020) it was proposed that although the human mind has the capacity to spatially organise information in either a left-to-right or right-to-left direction, when information is culturally mediated the cognitive system is more efficient in encoding and retrieval. This would suggest that in the current study, as both the narrow and wide condition were congruent to cultural reading direction, there could have either been a ceiling effect, or the efficiency of the cognitive system across all three conditions may not have allowed for a large enough difference in the spatial effect to be detected. It is also possible that the flexibility of the cognitive system could make it difficult to isolate what strategy for spatialisation is being used at an individual trial level.

To further explore spatialisation and address some of the limitations with the current study there are three proposals for future investigation that we could not address within the scope of this study. First, the experiment could be repeated using reverse presentation for the narrow and wide conditions both following a right-to-left direction. As it has already been established, a reverse SPoARC effect can be found with no spatial cost or interplay of reading direction; it could be suggested that in a right-to-left experimental condition the external presentation is more reliably recruited for internal spatial coding. Thus, employing a sole right-to-left experimental design could potentially resolve confounding effects from congruency with reading and writing direction that may occur in a left-to-right design. Second, it is possible to revert to using a between subjects’ design where participants are only exposed to one experimental condition. The design of the current study exposed each participant to all three conditions allowing for strong comparisons within subjects. While we implemented different trial blocks to allow for counterbalancing and reversal of instructions, exposure to all three conditions could have contributed a possible cross-over of the strategies used for spatialisation, again diluting the differences in SPoARC between each condition. Third, although we used a larger screen than was used in Guida, Abrahamse, and van Dijck (2020), it is still possible that the spatial distance between the narrow and wide conditions was not large enough or clear enough between the conditions to induce an effect. One way to further explore this is drastically increasing the screen size to be 100 cm wide, for example, to ensure there is no interference on the screen for any of the positions in either the narrow or wide condition and repeating the experiment. Overall, this study contributes evidence to support the robust findings for the SPoARC effect.

In summary, organisation of information in the mind is thought to occur through serial order coding employing mechanisms of positional tagging (Guida, Abrahamse, & van Dijck, 2020). Maintaining serial order is important in ensuring information stored in memory is organised in a way that can be coherently accessible (Lashley, 1951). Understanding the spatial element for serial order coding and more specifically how spatialisation occurs has implications for the efficient use of working memory (Majerus & Oberauer, 2020). As working memory correlates with our ability to learn, it is important to understand ways to maximise the efficiency of the limited capacity of the working memory system (Cowan, 2014). Practical implications for understanding spatial organisation are that it can inform ways of presenting information. If information is effectively presented this would minimise working memory load and lessen the effort needed in processing items, which would potentially improve our capacity for complex learning.

Finally, more broadly and beyond the scope of this study, further exploration of how information is spatialised using neural imaging techniques could also be an avenue for future investigation. While there is strong indication of a spatial element in organising information in the cognitive system using behavioural measures, less is known about the underlying neural mechanisms causing the behavioural responses within the SPoARC research. In a 2021 study using functional magnetic resonance imaging (fMRI), it was suggested that positional coding is mediated through spatial and motor control maps within the dorsal attentional system (Zhou et al., 2020). This could provide an alternative way to investigate nuances in the SPoARC effect when manipulating external presentation.

## Notes

1. Concerning the choice of the number of trials and the presentation time we were guided by two principles: first we wanted to create the conditions for the SPoARC to be detected, and second, we did not want the experiment to last too long (we wanted to stay below 50 mn to avoid participants’ fatigue). Our starting point was Guida, Abrahamse et al.’s (2020) paper, where they tested a SPoARC effect in sequences of five consonants. Items at encoding could be displayed (a) centrally, (b) from left to right (LR) across the screen or (c) from right to left (RL). Moreover, items were displayed at a pace of 1 s per item or 5 s per item. Results showed a classic left-to-right SPoARC effect in the central condition, while for the LR and RL conditions, SPoARC effects were congruent with the direction in which the items were displayed on the screen during encoding. Moreover, in the central condition, the SPoARC effect became larger with slower presentation rates (i.e., the slope was steeper in the 5 s condition). Conversely, the presentation rates did not influence the left-to-right and right-to-left conditions. Finally, in this experiment, the authors used in the 1 s per item presentation condition 54 trials and in the 5 s per item presentation condition 36 trials. When building the control condition in the present experiment (which correspond to the central condition in Guida, Abrahamse, & van Dijck, 2020), we thought about using 5 s in terms of presentation rate (as the SPoARC is larger with slower paces) with 36 trials, however that would create a too long experiment. So, we went down to 3 s and to give us more chances to observe the SPoARC effect, we used approximately the same number of trials as in Guida, Abrahamse, and van Dijck (2020) 1 s per item presentation condition. We preferred 56 compared with 54, because among the 56 probes (that come with 56 trials), 28 are positive probes (positive probes are the crucial part of the experiment) and 28 is divisible by 4 (we had four positions that were probed). At this point, we could have chosen to have 56 probes also for the narrow and the wide conditions, but it would have generated a too long experiment, so we went down one notch. Given that the crucial element is the number of positive probes, we choose the next divisible number by 4 below 28 that is 24, hence 48 probes/48 trials. Concerning the choice of using a 1s presentation for the narrow and the wide condition, again it was to keep the experiment as short as possible while being able to detect a SPoARC effect. We knew from Guida, Abrahamse, and van Dijck (2020) results that when the items are displayed from LR, the presentation time is not important (contrary to a central presentation), the SPoARC effect can be detected at slow or fast pace, so we used a fast pace because it shortens the experiment.
